# High Levels of CD244 Rather Than CD160 Associate With CD8^+^ T-Cell Aging

**DOI:** 10.3389/fimmu.2022.853522

**Published:** 2022-03-21

**Authors:** Xinyue Wang, Di Wang, Juan Du, Yuqing Wei, Rui Song, Beibei Wang, Shuang Qiu, Bei Li, Leidan Zhang, Yongqin Zeng, Hongxin Zhao, Yaxian Kong

**Affiliations:** ^1^ Peking University Ditan Teaching Hospital, Beijing, China; ^2^ Beijing Key Laboratory of Emerging Infectious Diseases, Institute of Infectious Diseases, Beijing Ditan Hospital, Capital Medical University, Beijing, China; ^3^ Beijing Institute of Infectious Diseases, Beijing, China; ^4^ National Center for Infectious Diseases, Beijing Ditan Hospital, Capital Medical University, Beijing, China; ^5^ Clinical and Research Center of Infectious Diseases, Beijing Ditan Hospital, Capital Medical University, Beijing, China; ^6^ Department of Laboratory, Beijing Ji Shui Tan Hospital, Beijing, China

**Keywords:** immune aging, aging, CD8+ T cells, CD160, CD244

## Abstract

Aging leads to functional dysregulation of the immune system, especially T cell defects. Previous studies have shown that the accumulation of co-inhibitory molecules plays an essential role in both T cell exhaustion and aging. In the present study, we showed that CD244 and CD160 were both up-regulated on CD8^+^ T cells of elderly individuals. CD244^+^CD160^-^ CD8^+^ T cells displayed the increased activity of β-GAL, higher production of cytokines, and severe metabolic disorders, which were characteristics of immune aging. Notably, the functional dysregulation associated with aging was reversed by blocking CD244 instead of CD160. Meanwhile, CD244^+^CD160^+^ CD8^+^ T cells exhibited features of exhaustion, including lower levels of cytokine, impaired proliferation, and intrinsic transcriptional regulation, compared to CD244^+^CD160^-^ population. Collectively, our findings demonstrated that CD244 rather than CD160 acts as a prominent regulator involved in T cell aging, providing a solid therapeutic target to improve disorders and comorbidities correlated to immune system aging.

## Introduction

Aging is accompanied by dysregulation of the immune system, which is characterized as immune aging and involves impaired immune responses and overwhelming inflammation (1). Immune aging contributes to high susceptibility to various age-related comorbidities, including malignancies, autoimmune diseases, and infectious diseases (2). In particular, infection of severe acute respiratory syndrome coronavirus 2 (SARS-CoV-2) was recently identified to associate with more severe respiratory illness and fatal pneumonia in the elderly and aged animal models, compared to young groups (3, 4).

Given that T cells are a prominent component of the immune system, T cell aging played a crucial role in the development of age-associated immune dysregulation (2). T cell exhaustion, another dominant dysfunctional state, differed from T cell senescence in phenotypic and functional features including surface molecules, cytokines, and transcriptional profiles (5–8). More importantly, T cell exhaustion was induced by persistent stimulation of T cells during chronic infection or tumor development, while T cell senescence could be caused by two major cellular mechanisms: replicative and premature senescence (9–11). Replicative senescence is a natural aging process that leads to shortening of telomere ends after multiple rounds of proliferation, whereas premature senescence is telomere-independent aging triggered by external factors such as cellular stress (12). Accordingly, while T cell exhaustion is thought to be reversible, T cell senescence was believed irreversible, which has been challenged in recent studies (13, 14). Moreover, T cell exhaustion is primarily regulated by inhibitory receptor-associated signaling pathways, while T cell senescence is mediated by mitogen-activated protein kinase (MAPK) signaling, which is involved in central signaling by targeting the cell cycle regulatory molecules p16, p21, and p53 (13–16).

Although senescent T cells were distinctly different from exhausted T cells in terms of generation, differentiation, signaling pathway, and molecular regulation, they shared several overlapping phenotypic and functional characteristics (16). Consistently, accumulated evidence demonstrated the involvement of increased co-inhibitory molecules in both T cell exhaustion and senescence. It is recognized that a panel of inhibitory receptors, including programmed cell death protein 1 (PD-1), cytotoxic T lymphocyte-associated antigen-4 (CTLA-4), T-cell immunoglobulin domain and mucin domain 3 (TIM-3), lymphocyte activation gene 3 (LAG-3), band T-lymphocyte attenuator (BTLA), T-cell immunoreceptor with Ig and ITIM domains (TIGIT), the natural killer cell receptor CD244 (also called 2B4), and the glycoprotein CD160, played a pivotal role in T cell exhaustion (17, 18). However, only a few of these molecules, such as TIGIT and TIM-3, were identified to associate with T cell senescence (19, 20).

Interestingly, a previous study reported increases in the expression of CD244 and CD160 on CD8^+^ T cells from aged mice (21). CD244 is a member of the signaling lymphocyte activated molecule (SLAM) subfamily of Ig superfamily receptors, which is expressed by NK cells and T cells. CD160, a glycosylphosphatidylinositol-anchored receptor, was identified on most NK cells and T cells (22–24). Despite being reported for their activating role in T cells, CD244 and CD160 mainly were recognized as inhibitory receptors to deliver negative signals, leading to T cell exhaustion during chronic infection and tumor progression (25, 26). However, whether CD244 and CD160 contributed to the immune aging of T cells has not been addressed. Here, we investigated the role of CD244 and CD160 in T-cell aging by using blood samples from healthy individuals. Our findings collectively demonstrated that upregulation of CD244 rather than CD160 is a crucial process related to aging of CD8^+^ T cells, whereas the importance of CD160 was confirmed in T cell exhaustion particularly.

## Materials and Methods

### Participants

This study was approved by the Committee of Ethics at Beijing Ditan Hospital, Capital Medical University, Beijing, China. All the human blood samples were collected with informed consent. Inclusion criteria targeted healthy volunteers aged 18-90 years (139 men and 186 women) recruited between February 2016 and December 2021. Gender was evenly distributed among all groups. A series of laboratory parameters including blood routine indexes, coagulation function, hepatic function, renal function, myocardial enzyme parameters, blood glucose, blood lipid, and tumor markers including alpha-fetoprotein (AFP), carcinoembryonic antigen (CEA), carbohydrate antigen (CA-199), CA-153, and CA-125 were considered in the exclusion criteria. Individuals were excluded from the study if they tested positive for human immunodeficiency virus (HIV) infection, hepatitis viral infections, systemic infection, connective tissue disease, cancer or abnormal laboratory indexes above. The Cytomegalovirus (CMV)-serostatus were not detected due to a high CMV seroprevalence, which is 40% during people’s first year of life (27) and ranges from 40 to 100% in adults (28–32).

### Isolation of Peripheral Blood Mononuclear Cells (PBMCs)

Peripheral blood samples were obtained from healthy controls, and PBMCs were separated by standard Ficoll-Paque gradient centrifugation according to the instructions of the manufacturer (Amersham Pharmacia Biotech, Sweden). Cells were cryopreserved in fetal bovine serum (FBS) (GIBCO, Grand Island, NY, USA) supplemented with 10% dimethylsulfoxide (DMSO) and stored in liquid nitrogen.

### Immunofluorescence Staining and Flow Cytometry Analysis

PBMCs from healthy subjects were resuspended in PBS buffer and were incubated with directly conjugated antibodies for 30 min at 4°C. The cells were washed with 1× PBS before flow cytometry analysis. Antibodies used included anti-human CD3-BV786 or CD3-BUV737, CD8-BV510 or CD8-BUV395, CD160-AF488, CD45RA-AF700, CD28-APC, CD95-PE, CD57-BV421, PD-1-BV711, TIM-3-BV650 (BD Biosciences, San Diego, CA, USA), CD4-APC-Fire750, CD244-PE-D594, CCR7-BV421, HLA-DR-AF700, CD38-BV421, CD28-BV711, CD27-BV650, KLRG-1-APC-Fire750, CD95-PE-CY7 (BioLegend, San Diego, CA, USA), TIGIT-PE-Cy7, LAG-3-APC (Ebioscience, San Diego, CA, USA) and the corresponding isotype controls. Data were acquired with the LSR Fortessa flow cytometer (BD Biosciences) and analyzed with FlowJo software version 10.5 (Tree Star, Ashland, OR, USA). More information about antibodies is listed in the **Supplementary Material** (**Table S2**).

### *In Vitro* Stimulation and Intracellular Staining

PBMCs were stimulated with anti-CD3/CD28 (5 µg/mL, Ebioscience) for 5 h in the presence of anti-CD107a BV421 (BioLegend) and Golgiplug (BD Biosciences). CD107a expression was measured as a marker of degranulation on CD8^+^ T cells after stimulation. The cells were surface-stained with CD3-BV786, CD4-APC-Fire750, CD8-BV421, CD244-PE-D594, CD160-AF488, and intracellularly stained with TNF-α-BV711, IL-2-BV650 (BioLegend), or IFN-γ-AF700 (Ebioscience) antibodies. For Ki67, perforin, Granzyme B, T-bet, or Eomes staining, PBMCs were surface-stained with CD3-BV786, CD4-APC-Fire750, CD8-BV421, CD244-PE-D594, CD160-AF488, and intracellularly stained with Granzyme B-AF700, T-bet-BV421 (BD Biosciences), Ki67-BV711, perforin-APC (BioLegend), or Eomes-PE-CY7 (Ebioscience) antibodies. A fixable viability dye eFluor^®^ 506 (Ebioscience) was used to label dead cells.

### Analysis of T-Cell Apoptosis

Apoptosis rates were assessed using an APC Annexin V apoptosis detection kit (BioLegend) following the manufacturer’s instructions, in combination with multiple markers for T cells (CD3, CD4, CD8, CD244, CD160). All samples were acquired and analyzed by flow cytometry.

### Measurement of Metabolic Parameters by Flow Cytometry

PBMCs were activated overnight by anti-CD3/CD28 beads (Gibco). Cells were stained with CD98-BUV395 (BD Biosciences) and CD71-FITC (BioLegend) antibodies, in combination with markers for T cells as mentioned above (30 min, 4°C). To evaluate glucose uptake, cells were cultured in RPMI-1640 glucose-free medium (Gibco) containing 10% FBS at 37°C for 30 min. Then, cells were washed and incubated with 100 μM 2-NBDG (Ebioscience) at 37°C for 30 min prior to fluorescence measurement by flow cytometry.

### RNA Extraction and Real-Time PCR

Total RNA, extracted from CD244^-^CD160^-^, CD244^+^CD160^-^ and CD244^+^CD160^+^ CD8^+^ T cells using RNeasy Micro Kit (Qiagen, United States), was applied to reverse-transcript into cDNA with the SuperScript IV First-Strand Synthesis System (Thermo Fisher Scientific, United States). Expression levels of metabolism-related genes were analyzed with LightCycler 480 Real-Time PCR (Roche Diagnostics GmbH, Mannheim, Germany) by applying SYBR Green. The sequences of primers were listed in **Supplementary Table 1**.

### Measurement of β-Gal Activity by Flow Cytometry

The activity of senescence-associated β-galactosidase (β-Gal), a marker of senescence, was analyzed with CellEvent senescence green flow cytometry assay kit (Thermo Fisher Scientific) as per the manufacturer’s instructions, in combination with markers for T cells as mentioned above. For fluorescence detection using flow cytometry, β-Gal activity was measured by the mean fluorescence intensity.

### CD244 and CD160 Blockade

CD8^+^ T cells were isolated from PBMCs by positive selection using EasySep™ human CD8 positive selection kit (StemCell Technologies, Vancouver, Canada). Purified cells were cultured at a concentration of 2 × 10^6^ cells/mL in a 96 well tissue culture plate and 10 µg/ml anti-human CD244 antibody (clone 999602; R&D systems), anti-human CD160 antibody (clone 688327; R&D systems) or isotype control was added to the culture medium. After 24 h of culture, CD8^+^ T cells were stimulated with anti-CD3/CD28 for 5 h in the presence of Golgiplug. Phenotypic staining and cytokine production were measured by flow cytometry.

### Statistical Analysis

The data are expressed as the mean ± standard deviation (SD). All data were analyzed using GraphPad7 (GraphPad Software, La Jolla, CA, USA). The normality of each variable was assessed using the Kolmogorov-Smirnov test. In cases of two normally distributed data, the comparison of variables was analyzed using unpaired or paired where specified, two-tailed Student’s t-tests for unpaired and paired data, respectively. A one-way ANOVA or a repeated-measures ANOVA followed by Tukey’s multiple comparisons test was performed for comparing more than two unpaired and paired samples, respectively. When the data were not normally distributed, the comparison of variables was analyzed with a Mann-Whitney U test or a Wilcoxon matched-pairs signed rank test for unpaired and paired data. For comparing more than two unpaired and paired samples, a Kruskal-Wallis test or a Friedman test with Dunn’s multiple comparisons test was used, respectively. The characteristics of the participant were compared using the Chi-square test (categorical variables) or the Kruskal-Wallis test (continuous variables). Pearson’s or Spearman’s correlation coefficients were performed to evaluate correlations for normally or non-normally distributed data, respectively. For all analyses, *P* < 0.05 was considered statistically significant.

## Results

### Age-Related Up-Regulation of CD244^+^, CD160^+^, and CD244^+^CD160^+^ CD8^+^ T Cells

**Figure 1:**
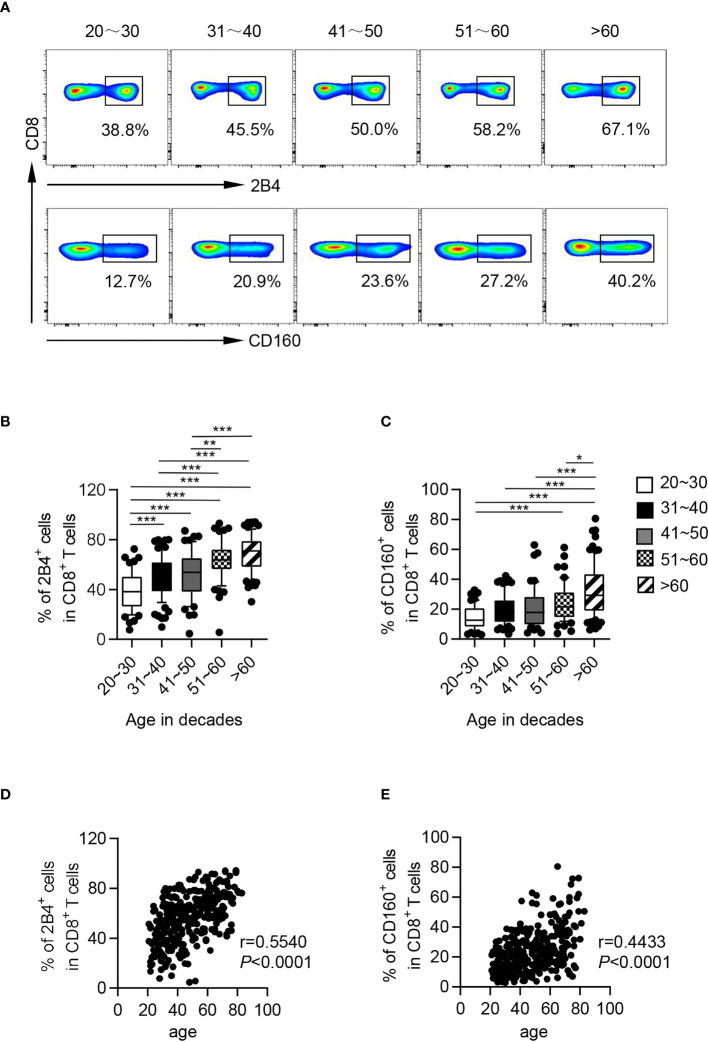
CD244 and CD160 on CD8^+^ T cells from healthy individuals were upregulated with age. Flow cytometry analysis of CD244 and CD160 expression was performed on PBMCs collected from healthy individuals of different ages. **(A)** Representative flow data show the expression of CD244 (up) and CD160 (down) gated on CD8^+^ T cells from five healthy individuals in different age groups. **(B, C)** Box plots of the percentage of CD244^+^ and CD160^+^ cells on CD8^+^ T cells from healthy individuals in different age groups (n = 52-84 each group). *P* values were obtained by one-way ANOVA followed by Tukey’s multiple comparisons test [CD244 (left)] or Kruskal-Wallis test followed by Dunn’s multiple comparisons test [CD160 (right)]. **(D, E)** Correlation analysis of age and CD244 **(D)**, CD160 **(E)** expression on CD8^+^ T cells from all healthy individuals. Spearman’s non-parametric test was used to test for correlations. *P < 0.05, **P < 0.01, ***P < 0.001.

To determine the potential role of CD244 and CD160 in T-cell aging, we performed a flow cytometric analysis of these two co-inhibitory molecules on T cells from 325 healthy adults (**Table 1**). The elderly (61-90 years old) showed significantly higher frequencies of CD244^+^ and CD160^+^ fractions among CD8^+^ T cells compared to young (21-30 and 31-40 years) and middle-aged individuals (41-50 and 51-60 years; **Figures 1A–C**). Meanwhile, CD244 and CD160 expression on CD4^+^ T cells were comparable in different age groups (**Figures S1A, B**). Correlation analysis further revealed that CD244^+^ and CD160^+^ cell frequencies among CD8^+^ T cells were remarkedly age-related (r = 0.5540, *P* < 0.0001 for CD244; r = 0.4433, *P* < 0.0001 for CD160; **Figures 1D, E**), whereas CD4^+^ T cells exhibited no correlation (r = 0.1502, *P* = 0.0067 for CD244; r = 0.1751, *P* = 0.0016 for CD160; **Figures S1C, D**).

**Table 1:** Characteristics of subjects in this study.

Parameters	Total (n = 325)	21~30 (n = 52)	31~40 (n = 78)	41~50 (n = 55)	51~60 (n = 56)	61-90 (n = 84)	*P* Value
**Gender**							
Male	139	20	41	24	23	31	0.3179
Female	186	32	37	31	33	53	
**Age, years**							
Median	46	26	36	44	55	68	<0.0001
IQR	35-61	24-29	34-38	42-47	53-58	64-73	

A total of 325 healthy adults were recruited, including 139 males and 186 females. Their median age was 46, and 52-84 adults were in every group. The Chi-square test demonstrated that the gender was balanced among all the groups (P = 0.3179). Age was described by median and interquartile range (IQR) and analyzed using Kruskal-Wallis test.

Based on expression of CD45RA, CCR7, CD28 and CD95, T cells are generally divided into six subsets: naïve T cells (T_N_, CCR7^+^CD45RA^+^CD28^+^CD95^-^), stem cell memory T cells (T_SCM_, CCR7^+^CD45RA^+^CD28^+^CD95^+^), central memory T cells (T_CM_, CCR7^+^CD45RA^-^CD28^+^CD95^+^), transitional memory T cells (T_TM_, CCR7^-^CD45RA^-^CD28^+^CD95^+^), effector memory T cells (T_EM_, CCR7^-^CD45RA^-^CD28^-^CD95^+^), and terminal effector T cells (T_TE_, CCR7^-^CD45RA^+^CD28^-^CD95^+^, **Figures S2A, B**) (33). Consistent with previous findings, we found decreased frequencies of CD8^+^ T_N_ cells during aging, along with an increase in most antigen-experienced populations, including T_SCM_, T_CM_, T_TM,_ and T_TE_ cells (**Figure S2C**). In addition, expression of CD244 and CD160 was mainly restricted to memory and effector T cells (**Figures S2D–G**). Nevertheless, CD244 and CD160 displayed differential changing trends in each CD8^+^ T cell subset. Although there was nearly no difference of CD160 expression on each CD8^+^ T cell subset among young and older individuals, the level of CD244 expression increased in almost each CD8^+^ T subset from the elderly compared to those in their counterparts from young and middle-aged individuals (**Figures S2D–G**, **S3**). This indicated that an elevated level of CD244 rather than CD160 is a general character of T cell aging.

**Figure 2:**
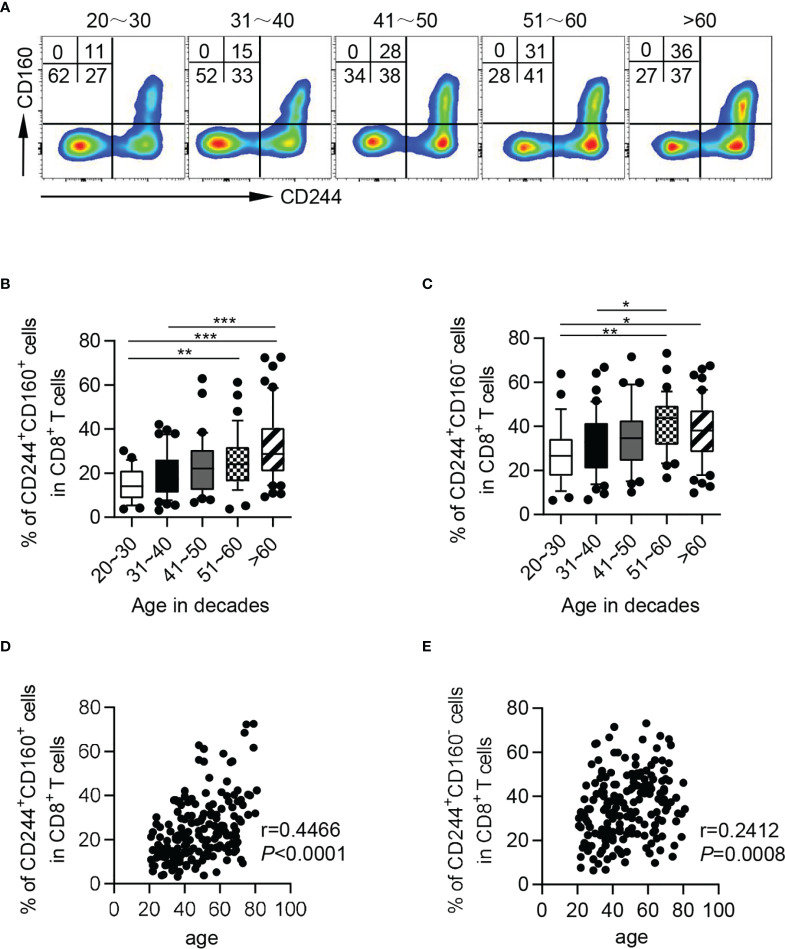
The frequencies of CD244^+^CD160^+^ and CD244^+^CD160^-^ CD8^+^ T cells in healthy individuals from different age groups. Flow cytometry analysis of frequencies of CD244^+^CD160^+^ and CD244^+^CD160^-^ CD8^+^ T cells from healthy donors of different ages. **(A)** Representative flow data show the frequencies of CD244^+^CD160^+^ and CD244^+^CD160^-^ cells gated on CD8^+^ T cells from five healthy donors in different age groups. **(B, C)** Box plots of the percentage of CD244^+^CD160^+^ **(B)** and CD244^+^CD160^-^ **(C)** cells on CD8^+^ T cells from healthy donors in different age groups (n = 27-50 each group). *P* values were obtained by one-way ANOVA followed by Tukey’s multiple comparisons test. **(D, E)** Correlation analysis of age and the percentage of CD244^+^CD160^+^ **(D)**, CD244^+^CD160^-^ **(E)** CD8^+^ T cells from all healthy individuals. Spearman’s non-parametric test were used to test for correlations. *P < 0.05, **P < 0.01, ***P < 0.001.

Interestingly, based on expression of CD244 and CD160, CD8^+^ T cells could be subdivided into three subsets: CD244^-^CD160^-^, CD244^+^CD160^+^ and CD244^+^CD160^-^. Nearly all CD160^+^ CD8^+^ T cells co-expressed CD244, thus CD244^-^CD160^+^ fraction was hardly observed (**Figure 2A**). We further found that CD244^+^CD160^+^ and CD244^+^CD160^-^ CD8^+^ T cells accumulated with aging. However, not an obvious increase in frequencies of CD244^+^CD160^-^ cells was observed in the elderly compared to the middle-aged individuals (**Figures 2B, C**). Consistently, correlation analysis showed a marked correlation of CD244^+^CD160^+^ fractions among CD8^+^ T cells with age (r = 0.4466, *P* < 0.0001), whereas no significant correlation was observed between CD244^+^CD160^-^ cells and age (r = 0.2412, *P* = 0.0008; **Figures 2D, E**).

### CD244^+^CD160^-^ CD8^+^ T Cells From the Elderly Exhibited Phenotypic and Functional Immune Aging

**Figure 3:**
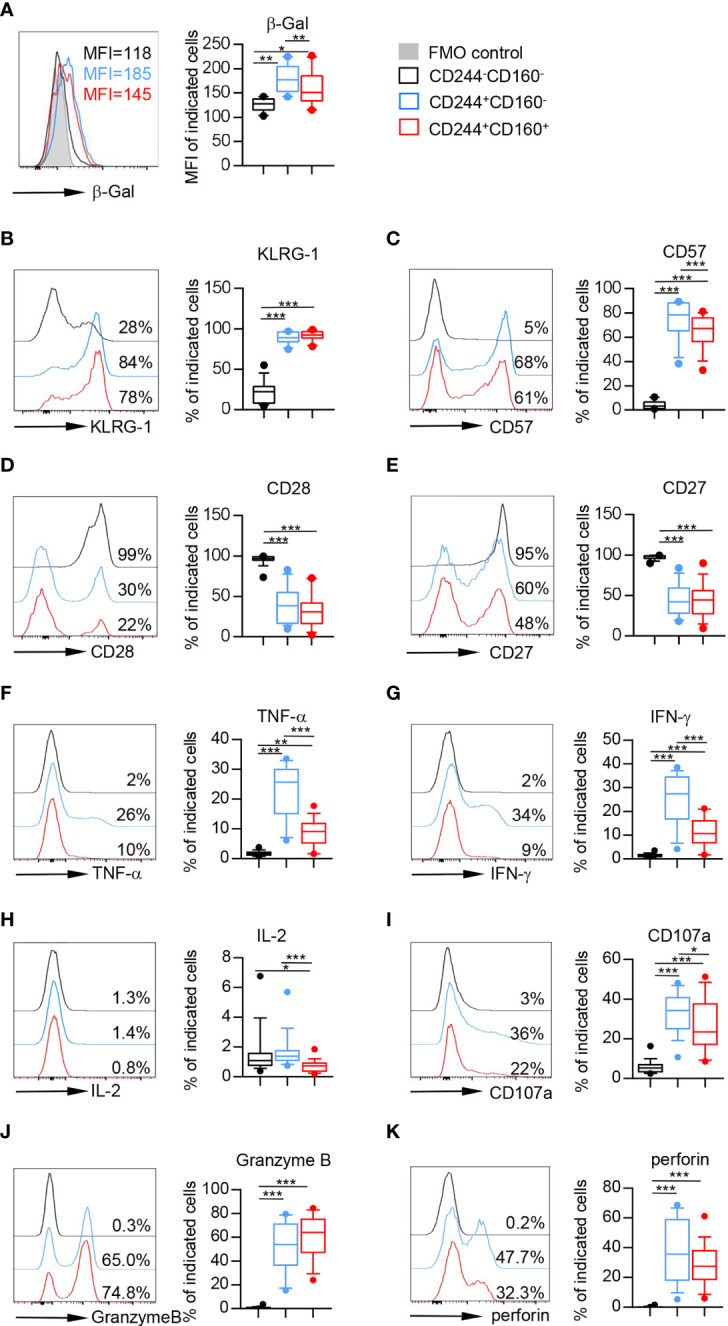
Flow cytometry analysis of senescent features on CD244^-^CD160^-^, CD244^+^CD160^-^ and CD244^+^CD160^+^ CD8^+^ T cells from the elderly. Flow cytometry analysis of senescence-associated markers on CD244^-^CD160^-^, CD244^+^CD160^-^ and CD244^-^CD160^+^ CD8^+^ T-cell subsets. **(A)** Representative histograms (left) and box plots (right) display the β-Gal activity measured by the mean fluorescence intensity (MFI) on CD244^-^CD160^-^, CD244^+^CD160^-^ and CD244^+^CD160^+^ CD8^+^ T cells (n = 10). *P* values were obtained by repeated-measures ANOVA followed by Tukey’s multiple comparisons test. **(B–E)** Representative histograms (left) and box plots (right) display the expression of KLRG-1 **(B)**, CD57 **(C)**, CD28 **(D)**, and CD27 **(E)** on CD244^-^CD160^-^, CD244^+^CD160^-^ and CD244^+^CD160^+^ CD8^+^ T cells from the elderly (61-90 years old, n = 15-17). *P* values were obtained by repeated-measures ANOVA followed by Tukey’s multiple comparisons test (KLRG-1, CD57, and CD27) or Friedman’s test followed by Dunn’s multiple comparisons test (CD28). **(F–H)** Intracellular staining for TNF-α, IFN-γ, and IL-2 on CD244^-^CD160^-^, CD244^+^CD160^-^ and CD244^-^CD160^+^ cells (gated with CD8^+^ T cells) from the elderly (61-90 years old, n = 17) after *in vitro* anti-CD3/anti-CD28 stimulation. Representative histograms (left) and box plots (right) for TNF-α **(F)**, INF-γ **(G)**, and IL-2 **(H)**, respectively. *P* values were obtained by repeated-measures ANOVA followed by Tukey’s multiple comparisons test (TNF-α and INF-γ) or Friedman’s test followed by Dunn’s multiple comparisons test (IL-2). **(I–K)** Expression of CD107a, Granzyme B, and perforin on CD244^-^CD160^-^, CD244^+^CD160^-^ and CD244^+^CD160^+^ CD8^+^ T cells from the elderly (61-90 years old, n = 17). Representative histograms (left) and box plots (right) of CD107a **(I)**, Granzyme B **(J)**, and perforin **(K)** expression. *P* values were obtained by repeated-measures ANOVA followed by Tukey’s multiple comparisons test (CD107a) or Friedman’s test followed by Dunn’s multiple comparisons test (Granzyme B and perforin). *P < 0.05, **P < 0.01, ***P < 0.001.

Senescent T cells were usually characterized by enhanced activity of senescence-associated beta-galactosidase (SA-β-Gal), high levels of killer cell lectin-like receptor subfamily G (KLRG-1) and CD57, and loss of CD28 and CD27 (34). To investigate whether CD244- and CD160-expressing CD8^+^ T cells in the elderly displayed senescent phenotypes, we examined the expression levels of the senescence-associated markers mentioned above. As shown in **Figure 3A**, the β-Gal activity was significantly up-regulated on CD244^+^CD160^-^ and CD244^+^CD160^+^ CD8^+^ T cells compared with that on the CD244^-^CD160^-^ subset. Accordingly, CD244^+^CD160^-^ and CD244^+^CD160^+^ CD8^+^ T cells from the elderly displayed higher levels of KLRG-1 and CD57 as well as lower levels of CD28 and CD27 than CD244^-^CD160^-^ fraction, indicating an impaired T cell immune response (**Figures 3B–E**). Surprisingly, CD244^+^CD160^-^ CD8^+^ T cells exhibited higher β-Gal activity and CD57 expression than the CD244^+^CD160^+^ subset (**Figures 3A, C**). By contrast, the expression levels of KLRG-1, CD28, and CD27 were comparable between CD244^+^CD160^-^ and CD244^+^CD160^+^ CD8^+^ T cells (**Figures 3B, D, E**).

As previously stated, aging-related increased inflammation is a common feature of immune aging (35). To determine the role of CD244 and CD160 in senescence-associated inflammatory responses of T cells, we then measured the cytokine release upon *in vitro* stimulation with anti-CD3 and anti-CD28 antibodies. CD244^+^CD160^-^ and CD244^+^CD160^+^ CD8^+^ T cells from older individuals produced significantly higher amounts of TNF-α and IFN-γ than the CD244^-^CD160^-^ population from the elderly (**Figures 3F, G**). Of note, CD244^+^CD160^+^ CD8^+^ T cells produced remarkedly lower levels of TNF-α and IFN-γ than the CD244^+^CD160^-^ subset (**Figures 3F, G**). Additionally, IL-2 production was decreased in CD244^+^CD160^+^ CD8^+^ T cells compared with that in CD244^-^CD160^-^ and CD244^+^CD160^-^ cells (**Figure 3H**). Furthermore, we evaluated the non-specific killing potential of CD244^-^CD160^-^, CD244^+^CD160^-^ and CD244^+^CD160^+^ CD8^+^ T cells from the elderly by measuring induced surface expression of CD107a and intracellular expression of Granzyme B and perforin. CD244^+^CD160^-^ and CD244^+^CD160^+^ CD8^+^ T cells from the elderly showed higher expression of CD107a, Granzyme B, and perforin than the CD244^-^CD160^-^ subset (**Figures 3I–K**). Notably, CD244^+^CD160^+^ CD8^+^ T cells exhibited reduced levels of CD107a and perforin compared with CD244^+^CD160^-^ CD8^+^ T cells (**Figures 3I, K**). Collectively, these results suggested that CD244^+^CD160^-^ CD8^+^ T cells were in a status of accelerated senescence compared to the CD244^+^CD160^+^ subset.

### CD244^+^ CD160^-^ and CD244^+^CD160^+^ CD8^+^ T Cells From the Elderly Showed Phenotypic and Functional Exhaustion, Which Remained Incomplete

**Figure 4:**
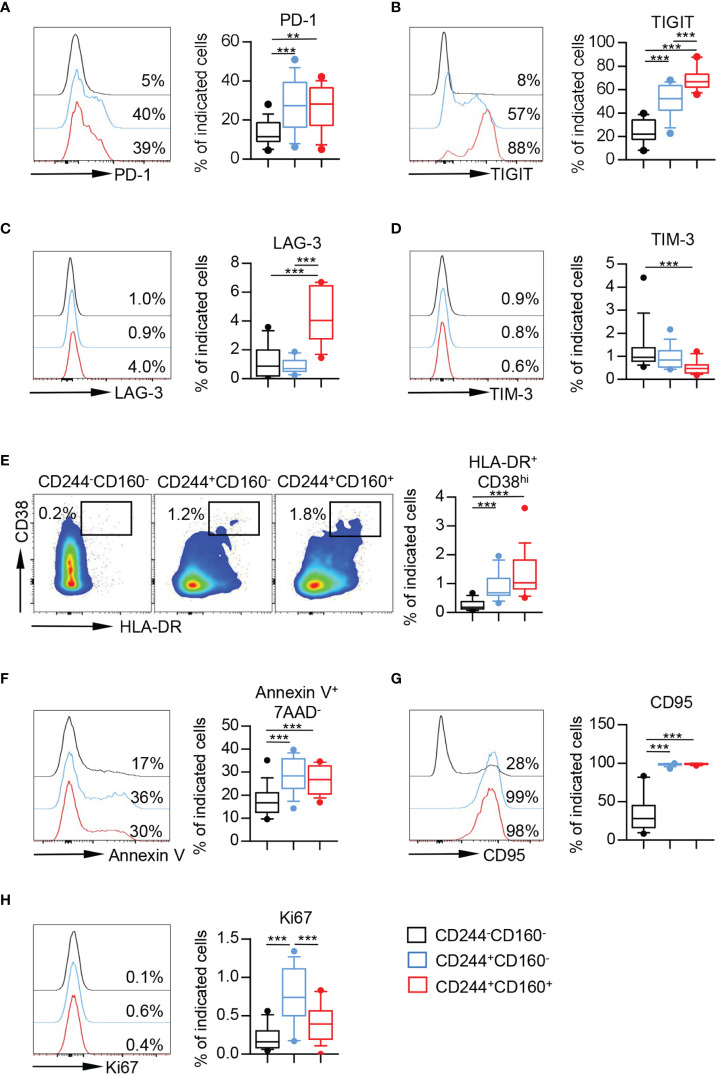
CD244^+^CD160^-^ and CD244^+^CD160^+^ CD8^+^ T cells from the elderly partially displayed exhaustion. **(A–E)** Flow cytometry analysis of the expression of PD-1 **(A)**, TIGIT **(B)**, LAG-3 **(C)**, TIM-3 **(D)**, and percentage of HLA-DR^+^CD38^hi^ cells **(E)** on CD244^-^CD160^-^, CD244^+^CD160^-^ and CD244^+^CD160^+^ CD8^+^ T cells (n = 11-17). Representative histograms or flow data (left), and box plots (right) display the expression of the above receptors on CD244^-^CD160^-^, CD244^+^CD160^-^ and CD244^+^CD160^+^ cells (gated with CD8^+^ T cells). *P* values were obtained by repeated-measures ANOVA followed by Tukey’s multiple comparisons test (PD-1, TIGIT, and LAG-3) or Friedman’s test followed by Dunn’s multiple comparisons test (TIM-3 and HLA-DR^+^CD38^hi^). **(F–H)** Percentage of apoptotic cells (Annexin V^+^7AAD^-^) **(F)** and expression of CD95 **(G)** and Ki67 **(H)** on CD244^-^CD160^-^, CD244^+^CD160^-^ and CD244^+^CD160^+^ CD8^+^ T cells from the elderly (61-90 years old, n = 15-17). Representative histograms (left) and box plots (right) display the percentage of apoptotic cells (Annexin V^+^ 7AAD^-^) **(F)** and expression of CD95 **(G)** and Ki67 **(H)** on CD244^-^CD160^-^, CD244^+^CD160^-^ and CD244^+^CD160^+^ cells (gated with CD8^+^ T cells). *P* values were obtained by repeated-measures ANOVA followed by Tukey’s multiple comparisons test (Annexin V^+^7AAD^-^ and CD95) or Friedman’s test followed by Dunn’s multiple comparisons test (Ki67). **P < 0.01, ***P < 0.001.

To further characterize the phenotype of CD244- and CD160-expressing CD8^+^ T cells from aging individuals, we compared the expression of several co-inhibitory molecules on CD244^-^CD160^-^, CD244^+^CD160^-^ and CD244^+^CD160^+^ fractions of CD8^+^ T cells. The levels of PD-1 and TIGIT were significantly higher on CD244^+^CD160^-^ and CD244^+^CD160^+^ CD8^+^ T cells than those on CD244^-^CD160^-^ CD8^+^ population (**Figures 4A, B**). Moreover, CD244^+^CD160^+^ CD8^+^ T cells expressed higher levels of TIGIT than CD244^+^CD160^-^ cells (**Figure 4B**). We also observed increased expression of LAG-3 in CD244^+^CD160^+^ CD8^+^ T cells compared to CD244^-^CD160^-^ and CD244^+^CD160^-^ cells (**Figure 4C**). Meanwhile, there was no significant difference in LAG-3 expression between CD244^-^CD160^-^ and CD244^+^CD160^-^ subsets (**Figure 4C**). In addition, we found lower expression of TIM-3 on CD244^+^CD160^+^ cells compared to CD244^-^CD160^-^ cells (**Figure 4D**).

Given that T cell exhaustion was regarded as a result of excessive T cell activation induced by persistent antigen stimulation, we then assessed the activation status of these T cell subsets by measuring co-expression of HLA-DR and CD38. The results performed higher percentages of HLA-DR^+^CD38^hi^ cells in CD244^+^CD160^-^ and CD244^+^CD160^+^ fractions than in CD244^-^CD160^-^ CD8^+^ T cells (**Figure 4E**). In addition, CD244^+^CD160^+^ CD8^+^ T cells from the elderly showed slight elevation of HLA-DR^+^ CD38^hi^ cells compared to the CD244^+^CD160^-^ subset; however, no statistically significant difference was detected (**Figure 4E**).

We next assessed the susceptibility to apoptosis of CD244^-^CD160^-^, CD244^+^CD160^-^ and CD244^+^CD160^+^ CD8^+^ T cells from older individuals. CD244^+^CD160^-^ and CD244^+^CD160^+^ CD8^+^ T cells in the elderly exhibited increased frequencies of Annexin-V^+^7AAD^-^ and CD95^+^ cells compared to the CD244^-^CD160^-^ population, indicating a high susceptibility to apoptosis (**Figures 4F, G**). Moreover, we found a significant increase of Ki67 expression in CD244^+^CD160^-^ and CD244^+^CD160^+^ CD8^+^ T cells in comparison with CD244^-^CD160^-^ subset from the elderly (**Figure 4H**). Of interest, CD244^+^CD160^+^ CD8^+^ T cells displayed lower levels of Ki67 than CD244^+^CD160^-^ cells (**Figure 4H**). Collectively, these data indicated that CD244^+^CD160^-^ and CD244^+^CD160^+^ CD8^+^ T cells from the elderly were in a status of exhaustion by displaying a high expression of co-inhibitory molecules, enhanced susceptibility to apoptosis while retaining their capacity of proliferation. More importantly, CD244^+^CD160^+^ CD8^+^ T cells appeared to be more exhausted in phenotypic and functional characteristics than CD244^+^CD160^-^ cells.

### CD244^+^CD160^-^ CD8^+^ T Cells Exhibited Metabolic Disorders

**Figure 5:**
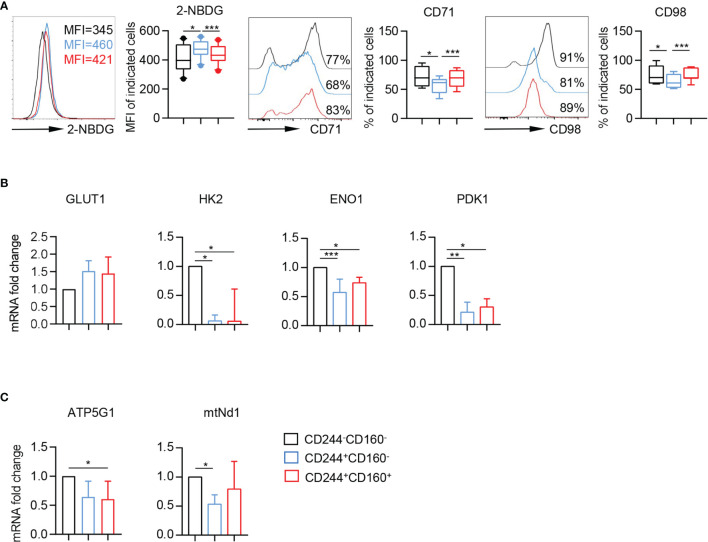
Enhanced glucose uptake and reduced expression of metabolism-associated genes in CD244^+^CD160^-^ CD8^+^ T cells. **(A)** Representative histograms (left) and box plots (right) of 2-NBDG uptake or CD71 and CD98 expression in CD244^-^CD160^-^, CD244^+^CD160^-^ and CD244^+^CD160^+^ CD8^+^ T cells (n = 7-10). *P* values were obtained by repeated-measures ANOVA followed by Tukey’s multiple comparisons test. **(B, C)** Differential effects of CD244 and CD160 on markers of the glycolysis and oxidative phosphorylation in CD8^+^ T cells from older subjects (n = 5-7). Real-time quantitative PCR analysis of transcript levels of **(B)** glycolytic enzymes (GLUT1, HK2, ENO1, and PDK1), and **(C)** oxidative phosphorylation (ATP5G1 and mtNd1) in CD8^+^ T cells after 4 h of culture in the presence of plate-bound anti-CD3 and anti-CD28 (10 µg/mL). *P* values were obtained by Friedman’s test followed by Dunn’s multiple comparisons test. *P < 0.05, **P < 0.01, ***P < 0.001.

Nutrient uptake and glucose metabolism are crucial in the immune responses of T cells. To further understand the metabolic regulation of CD244- and CD160-expressing CD8^+^ T cells, we analyzed three commonly-used metabolic parameters, including 2-[N-(7-nitrobenz-2-oxa-1, 3-diaxol-4-yl) amino]-2-deoxyglucose (2-NBDG) uptake, CD71 (transferrin receptor), and CD98 (amino-acid transporter). We found that the glucose uptake ability of CD244^+^CD160^-^ CD8^+^ T cells was significantly increased, compared to CD244^-^CD160^-^ and CD244^+^CD160^+^ CD8^+^ T cells (**Figure 5A**). On the contrary, these CD244^+^CD160^-^ CD8^+^ T cells showed significantly lower levels of CD71 and CD98 than CD244^-^CD160^-^ and CD244^+^CD160^+^ subsets (**Figure 5A**). Additionally, CD244^+^CD160^+^ CD8^+^ T cells had comparable glucose uptake and expression levels of nutrient receptors (CD71 and CD98) with CD244^-^CD160^-^ fraction. We next examined the expression levels of several metabolism-associated genes in CD244^-^CD160^-^, CD244^+^CD160^-^ and CD244^+^CD160^+^ CD8^+^ T cells from the elderly by RT-PCR. In agreement with glucose uptake, glucose transporter GLUT1 was also up-regulated in CD244^+^CD160^-^ CD8^+^ T cells than CD244^-^CD160^-^ and CD244^+^CD160^+^ CD8^+^ T cells (**Figure 5B**). Glycolysis-related genes (HK2, ENO1, and PDK1) were significantly down-regulated in both CD244^+^CD160^-^ and CD244^+^CD160^+^ cells than in CD244^-^CD160^-^ CD8^+^ T cells (**Figure 5B**). Moreover, analysis of two genes involved in oxidative phosphorylation showed a significant down-regulation of ATP5G1 in CD244^+^CD160^+^ CD8^+^ T cells and a reduction of mtNd1 in CD244^+^CD160^-^ CD8^+^ T cells compared to CD244^-^CD160^-^ population (**Figure 5C**). In addition, there was no significant difference in expression levels of glycolysis and oxidative phosphorylation-related genes between CD244^+^CD160^-^ and CD244^+^CD160^+^ fractions (**Figures 5B, C**). These results showed impaired glycolysis but enhanced glucose uptake in CD244^+^CD160^-^ CD8^+^ T cells, suggesting an obvious disorder of glucose metabolism during immune aging.

### CD244^+^CD160^-^ CD8^+^ T Cells Comprised a High Frequency of T-bet^hi^Eomes^dim^ Cells, Which Retained Some Residual T Cell Functions

**Figure 6:**
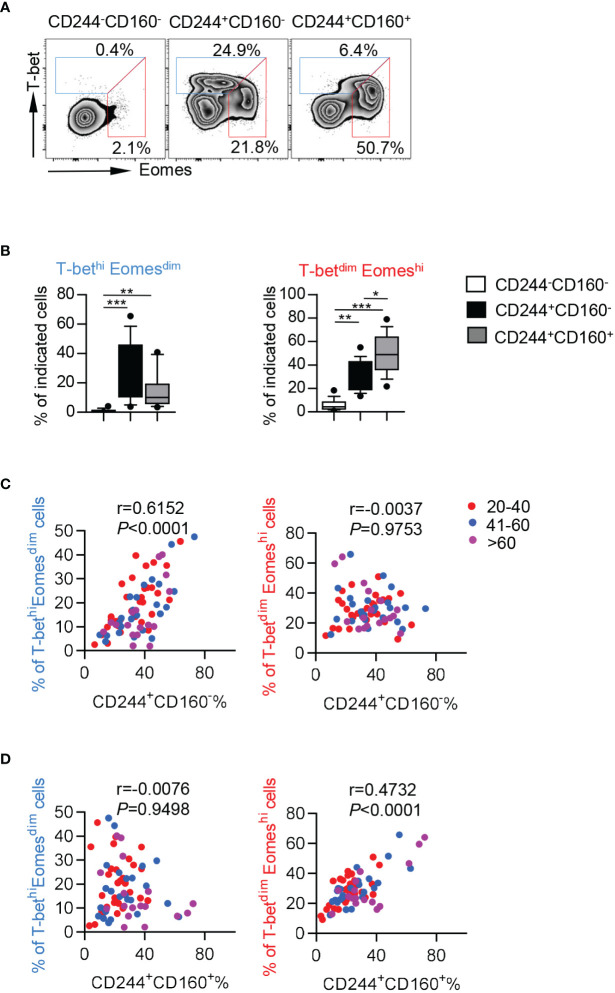
CD244^+^CD160^-^ CD8^+^ T cells from the elderly exhibited elevated T-bet^hi^Eomes^dim^ cells while CD244^+^CD160^+^ CD8^+^ T cells comprised a high number of T-bet^dim^Eomes^hi^ cells. **(A, B)** Representative flow data **(A)** and box plots **(B)** of the percentage of T-bet^dim^Eomes^hi^ and T-bet^hi^Eomes^dim^ cells on CD244^-^CD160^-^, CD244^+^CD160^-^ and CD244^+^CD160^+^ CD8^+^ T cells in the elderly (61-90 years old, n = 17). *P* values were obtained by Friedman’s test followed by Dunn’s multiple comparisons test. **(C, D)** Correlation analysis of the percentage of CD244^+^CD160^-^ and CD244^+^CD160^+^ cells and the frequencies of T-bet^hi^Eomes^dim^ and T-bet^dim^Eomes^hi^ cells on CD8^+^ T cells of all ages. Spearman’s non-parametric test was used to test for correlations. *P < 0.05, **P < 0.01, ***P < 0.001.

To further investigate the intrinsic regulation of CD244- and CD160-expressing CD8^+^ T cells, we assayed the expression of T-bet and Eomesodermin (Eomes), which are two homologous T-box transcription factors and cooperate to modulate both functional and dysfunctional CD8^+^ T cell responses. T-bet^dim^Eomes^hi^ CD8^+^ T cells were identified as a terminal exhausted population, whereas T-bet^hi^Eomes^dim^ cells retained some residual effector functions (36). We observed higher percentages of both T-bet^hi^Eomes^dim^ and T-bet^dim^Eomes^hi^ cells in CD244^+^CD160^-^ and CD244^+^CD160^+^ CD8^+^ T cells than in CD244^-^CD160^-^ cells from the elderly (**Figures 6A, B**). Notably, CD244^+^CD160^+^ CD8^+^ T cells contained a lower frequency of T-bet^hi^Eomes^dim^ cells but a higher percentage of T-bet^dim^Eomes^hi^ cells than CD244^+^CD160^-^ CD8^+^ T cells, suggesting a greater loss of T cell function (**Figure 6B**). Consistently, the frequency of CD244^+^CD160^-^ CD8^+^ T cells was significantly correlated with T-bet^hi^Eomes^dim^ cells (r = 0.6152, *P* < 0.0001) but not with T-bet^dim^Eomes^hi^ cells (r = 0.0037, *P* = 0.9753; **Figure 6C**). Meanwhile, correlation analysis revealed a positive correlation between the percentage of CD244^+^CD160^+^ CD8^+^ T cells and T-bet^dim^Eomes^hi^ frequency (r = 0.4732, *P* < 0.0001; **Figure 6D**). Collectively, these data suggested that the fate of CD8^+^ T cells was determined to diverse directions by CD244 and CD160. CD244^+^CD160^-^ CD8^+^ T cells contained a high number of T-bet^hi^Eomes^dim^ cells with remaining functions, whereas CD244^+^CD160^+^ CD8^+^ T cells mainly comprised terminal exhausted T-bet^dim^Eomes^hi^ cells.

### CD244 But Not CD160 Blockade Partly Reverses the Age-Associated Phenotype of CD8^+^ T Cells

**Figure 7:**
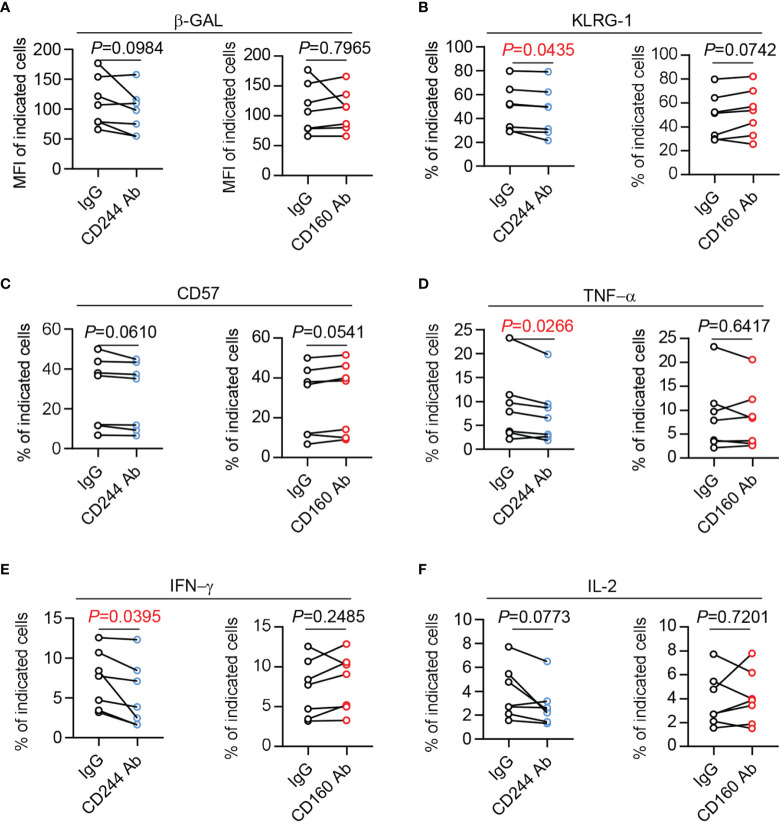
The senescent status and cytokine production of CD8^+^ T cells could be reversed by CD244 but not CD160 blockade. Purified CD8^+^ T cells from healthy donors (n = 7) were cultured with antagonist anti-CD244, anti-CD160 antibody, or isotype IgG at a concentration of 10 µg/mL. After culturing *in vitro* for 24h, the activity of β-Gal **(A)**, KLRG-1 **(B)**, CD57 **(C)**, TNF-α **(D)**, IFN-γ **(E)**, and IL-2 **(F)** expression on CD8^+^ T cells was measured by flow cytometry. Representative plots of the above markers in CD8^+^ T cells. *P* values were obtained by paired t-test.

To study the direct effect of CD244 and CD160 in T cell dysfunction, we performed CD244 and CD160 blockade experiments using anti-human CD244 or CD160 antibodies. We found a trend towards down-regulated β-Gal activity and CD57 expression in CD8^+^ T cells upon CD244 blockade. However, the trend did not reach statistical significance (**Figures 7A, C**). Of note, a significant decrease of KLRG-1 expression was observed in CD8^+^ T cells by blocking CD244 (**Figure 7B**). Moreover, CD8^+^ T cells produced fewer cytokines, including TNF-α, IFN-γ,and IL-2, after the blockade of CD244 (**Figures 7D–F**). Oppositely, we found that CD160 blockade could not reverse senescent phenotypes and decrease cytokine release in CD8^+^ T cells (**Figures 7A–F**). Thus, CD244 rather than CD160 plays a more critical role in the regulation of T cell aging.

## Discussion

Previous studies have noted that both T cell exhaustion and senescence coexisted in patients with chronic viral infections and various types of cancers, which led to elevated susceptibility to viral infections and malignant tumors (37, 38). In the present study, we further demonstrated remarkable enrichment and coexistence of senescent and exhausted T cells in elderly individuals. It is addressed that exhausted and senescent T cells share several similar characteristics of phenotype and function, including impaired cytotoxic activity, defective proliferative capacity, and increased cell cycle arrest. However, senescent T cells also showed unique signatures from exhausted cells, consisting of high expression of SA-β-Gal and senescence-associated secretory phenotype (SASP) (39, 40). Although upregulation of CD244 and CD160 were both observed in the elderly, levels of CD244 displayed a greater value of correlation with age than CD160, which was supported by the differential changing trends of CD244 and CD160 in each CD8^+^ T cell subset. In particular, higher frequency of CD244^+^ CD8^+^ T cells from the elderly compared with that from young subjects were observed in T cell at almost each differentiation stage. In contrary, no significant differences of CD160 expression were observed in most of CD8^+^ T subsets between the young and the elderly. Thus, our comprehensive phenotypic studies demonstrated that the upregulation of CD244 on CD8^+^ T cells from elderly subjects were nearly independent of differentiation status.

Moreover, only CD244^+^CD160^-^ CD8^+^ T cells were identified as senescent population, manifested by increased activity of β-GAL, higher production of cytokines, and severe metabolic disorders. Importantly, their functional dysregulation associated with aging could be reversed by blocking CD244 instead of CD160. Meanwhile, CD244^+^CD160^+^ CD8^+^ T cells exhibited characteristics of exhaustion, such as lower levels of cytokine, impaired proliferation, and typic transcriptional regulation, compared to CD244^+^CD160^-^ population. These data suggested that CD244 rather than CD160 elucidate a potential mechanism of T cell aging, whereas CD160 is more likely to involve in age-associated exhaustion of T cells.

Unlike CD160-triggered exhausted T cells, CD244^+^CD160^-^ CD8^+^ T cells secreted elevated proinflammatory cytokines, and expressed high levels of CD107a and intracellular perforin. Recent studies demonstrated that increased baseline inflammation in older individuals, called inflamm-aging, is a common feature of immune aging (41). This phenotype, which is also referred to as SASP, provides a strong link between aging and inflammation. In accordance with our findings, senescent T cells expressed a great number of secreted proteins (34). Of note, recent studies indicated that the presence of excessive inflammation could inhibit antigen-specific immunity in both animals and humans infected with viruses such as the influenza virus (42). Thus, overwhelming inflammation and consequent impaired viral clearance in the elderly infected with SARS-CoV-2 are thought to underlie the devastating morbidity and mortality associated with aging (43). Consistently, increased levels of both CD244 and CD160 in patients infected SARS-CoV-2 and other viruses indicated that T cell aging occurred as well as exhaustion in viral infection (44, 45). A thorough understanding of how aging impacts the immune response of the elderly would be helpful to determine the effective treatment for severe COVID-19 patients.

An interesting finding in the present study was that senescent CD244^+^CD160^-^ CD8^+^ T cells showed high expression of Annexin V and CD95, indicating an enhanced sensitivity to apoptosis. In contrast to induced apoptosis in exhausted T cells, the traditional concept of cellular senescence indicated resistance to apoptosis for senescent fibroblasts and tumor cells (46, 47). However, in line with our findings, several studies identified senescence as a tumor-suppressive mechanism that triggers apoptosis in different types of cells (48, 49). It appears that both cell apoptosis and senescence are preceded by earlier events of massive DNA damage, endoplasmic reticulum stress, and reactive oxygen species (ROS). Numerous tumor suppressors, including p53, Rb, and ING, could be involved in the processes and led to the induction of both apoptosis and senescence (50, 51). Some microRNAs such as miR-200c and other miR-200 family members were also found to induce oxidative stress-associated apoptosis and senescence of endothelial cells (52). Of interest, it was revealed that cell senescence is accompanied by apoptosis during embryonic development and organ morphogenesis (53). Thus, considering that most classical conclusions about senescence are based on *in vitro* experiments using non-physiological stresses, it is unsurprising for the contradictory view on the relationship between senescence and apoptosis. Here, we provided evidence linking CD244-associated T-cell aging to apoptosis. The similar patterns of apoptosis and senescence implied crosstalk between both processes and needed to be further investigated.

Despite being considered as classical co-inhibitory molecules, both CD244 and CD160 signaling can lead to dual functions affecting cytotoxicity and inhibition of T cells. However, the causes for these opposing functions of CD244 and CD160 could be explained with distinct interpretations. CD244 signaling can result in both inhibition and activation of T cell function, depending on the expression density of CD244 and diverse intracellular adaptor molecules (24). Instead, CD160 was reported to have a co-stimulatory role upon binding to MHC-I ligands or mediate a co-inhibitory effect upon binding to herpes virus entry mediator (HVEM). What is more, HVEM could serve as a receptor in addition to a ligand for its binding partners, leading to proinflammatory or inhibitory signals (54). Thus, it is not surprising that CD244 and CD160 play different roles in T cell aging and exhaustion. Further structural and signaling dissection of CD244 and CD160 would be helpful to clarify the conclusions drawn in T cell aging.

Of surprise, aged CD244^+^CD160^-^ CD8^+^ T cells showed a higher capacity of proliferation than CD244^-^CD160^-^ fraction, which is contradictory to the classical definition that cellular senescence is traditionally considered as a state of irreversible growth arrest. It is worth noting that most aged naive and memory T cells do not present the phenotypic features of senescent cells, such as increased size, increased lysosomal content and function, and vacuolated and granular morphology (13, 55). Most importantly, most aged naive and memory T cells can proliferate when properly activated. In fact, aged naive CD4^+^ T cells from rheumatoid arthritis patients divide faster than those in age-matched healthy controls, even though they showed accelerated aging (56). Additionally, given that memory T cells displayed prominent stem cell-like features, including long-term lived and underwent self-renewal, maintenance in the G0 phase of the cell cycle is crucial to support the lifespan of memory T cells (57). In this regard, aged CD244^+^CD160^-^ CD8^+^ T cells with high proliferation are not able to stay in the G0 phase and enter the next cell cycle stage, lose the ability to self-renew, and eventually undergo their immunosenescent fate.

It was addressed that two transcription factors, T-bet and Eomes, played crucial roles in the control of CD8^+^ T cell fate decisions to exhaustion or memory (58). T-bet and Eomes cooperate in many aspects; however, their expression is somewhat reciprocal. In fact, the long-term fate of CD8^+^ T cell functionality and differentiation was sensitively regulated by the relative ratio of T-bet and Eomes. For example, the loss of polyfunctional HIV-specific CD8^+^ T cells was reported to associate with upregulation of Eomes as well as decreased expression levels of T-bet (59). Of note, T-bet^hi^Eomes^dim^ CD8^+^ T cells were predominant in the early effector stage and then lost T-bet expression, impaired function, and converted to T-bet^dim^Eomes^hi^ subset after persistent antigen stimulation (36). In this aspect, T-bet^hi^Eomes^dim^ cells still retained some functional capacities, whereas the T-bet^dim^Eomes^hi^ terminal population expressed higher levels of inhibitory receptors and exhibited poor functional abilities (60). In the current study, we observed an elevated percentage of T-bet^dim^Eomes^hi^ cells and decreased proportion of T-bet^hi^Eomes^dim^ in aged CD244^+^CD160^-^ CD8^+^ T cells compared with the exhausted CD244^+^CD160^+^ population. Moreover, the percentage of CD244^+^CD160^-^ cells was significantly correlated to T-bet^hi^Eomes^dim^ cell frequency, whereas the CD244^+^CD160^+^ population showed a positive correlation with T-bet^dim^Eomes^hi^ cells. These results provided evidence for the notion that aged CD244^+^CD160^-^ CD8^+^ T cells still retain part of effector functions compared to exhausted CD244^+^CD160^+^ cells, which lost most of the functional characteristics.

Glycolysis, oxidative phosphorylation (OXPHOS), fatty acid oxidation (FAO), and amino acid metabolism were reported to generate energy in T cells (61, 62). Maintaining a specific metabolic pattern is essential for T cells to sustain their functions at different stages. When T cells were aged, metabolic alterations or dysfunction in the T cell subsets remained complicated. CD244^+^CD160^-^ CD8^+^ T cells showed a higher ability of glucose uptake and transport than CD244^+^CD160^+^ and CD244^-^CD160^-^ CD8^+^ T cells, indicating that more glucose was taken into cells. Davenport B et al. also found elevated 2-NDBG uptake and GLUT1 expression in aged memory CD8 T cells when in the steady-state (63). Although more glucose was taken into cells, glycolysis (HK2, ENO1, and PDK1) and OXPHOS (mtNd1) were significantly suppressed in aged CD244^+^CD160^-^ CD8^+^ T cells, which was in accordance with the study of young and aged T cells in mice. They found that aged T cells exhibited defects in glycolysis and OXPHOS after TCR-driven activation (64). However, several studies reported that elevated glycolysis was associated with senescence triggered by oncogenic or genotoxic stress in fibroblast cells (65). The different states of glycolysis in senescent cells might be a result of the distinct ways to induce aging (natural aging or stress-induced aging) in various cell types. The discrepancy highlights the importance of understanding metabolic regulation in response to natural aging in T cell senescence. In addition, amino-acid transporter CD98 was known to promote the uptake of glutamine from plasma, which could maintain homeostasis of redox state and prevent oxidative stress (66). Here, CD98 was significantly decreased in CD244^+^CD160^-^ CD8^+^ T cells, indicating that loss of redox balance and oxidative stress might induce immune aging in these cells. However, we did not further analyze the metabolic parameters in the same cell subsets at different ages due to limited cell numbers. More detailed metabolic dissection of the same T subsets from each age group would be helpful to clarify the conclusions.

There are some limitations in the present study, including a lack of further phenotypic and functional analysis in T cell subsets, low sensitivity of flow-based method for detecting SA-β-Gal activity, and limited indicators of immunosenescence. Other indicators of classical senescence such as p16, p53-p21 signaling pathway and telomere attrition could be considered in our future study, providing a benefit for elucidating the unique mechanisms of immune aging.

In summary, our study demonstrated that CD244 instead of CD160 is an important immune regulator involved in the process of T cell aging, making it an effective therapeutic target to improve age-related immune disorders and comorbidities.

## Data Availability Statement

The original contributions presented in the study are included in the article/**Supplementary Material**. Further inquiries can be directed to the corresponding authors.

## Ethics Statement

The studies involving human participants were reviewed and approved by the Committee of Ethics at Beijing Ditan Hospital. The patients/participants provided their written informed consent to participate in this study.

## Author Contributions

XW, DW, and JD performed the experiments and analyzed the data. YW, RS, BW, SQ, BL, LZ, and YZ collected samples, and performed the experiments. HZ participated in the critical review of the manuscript and revised the manuscript. YK designed the experiments, analyzed the data and wrote the manuscript. All authors contributed to the article and approved the submitted version.

## Funding

This work was supported by National Natural Science Foundation of China (81971307, 82171548), Beijing Municipal Natural Science Foundation for Distinguished Young Scholars (JQ21023), Beijing Municipal Administration of Hospitals’ Ascent Plan (DFL20191802), and Beijing Municipal Administration of Hospitals Clinical Medicine Development of Special Funding Support (ZYLX202126).

## Conflict of Interest

The authors declare that the research was conducted in the absence of any commercial or financial relationships that could be construed as a potential conflict of interest.

## Publisher’s Note

All claims expressed in this article are solely those of the authors and do not necessarily represent those of their affiliated organizations, or those of the publisher, the editors and the reviewers. Any product that may be evaluated in this article, or claim that may be made by its manufacturer, is not guaranteed or endorsed by the publisher.
