# Supplementary material for: High Levels of CD244 Rather Than CD160 Associate With CD8+ T-Cell Aging
Source: Front Immunol. 2022 Mar 21;13:853522. doi: 10.3389/fimmu.2022.853522 (PMC8977780; doi:10.3389/fimmu.2022.853522)
Supplement: Supplementary file 1 [file DataSheet_1.pdf]

Supplementary Material

Supplementary Figures

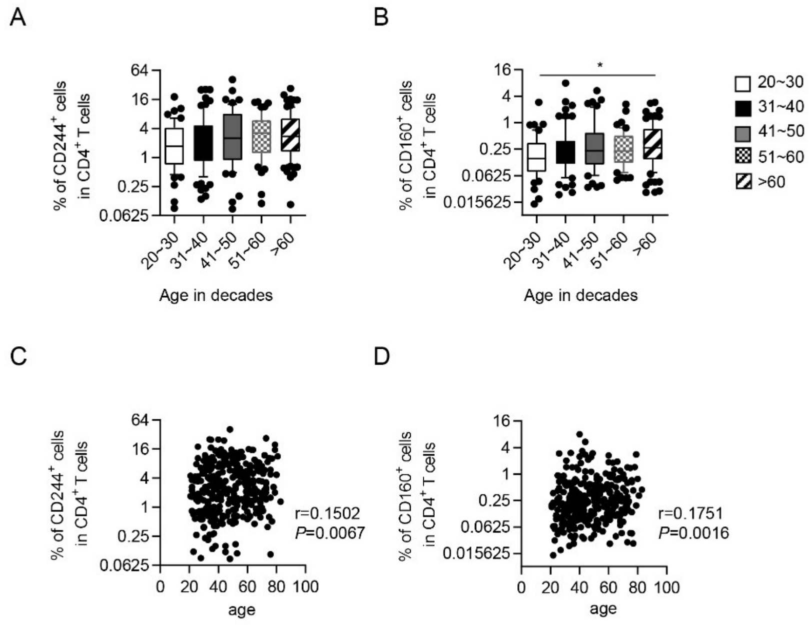

Supplementary Figure 1. **Expression of CD244 and CD160 on CD4+ T cells of healthy individuals from different age groups**

(A-B) Box plots of the percentage of CD244+ and CD160+ cells on CD4+ T cells from healthy donors in different age groups (n = 52-84 each group). *P* values were obtained by Kruskal-Wallis test followed by Dunn's multiple comparisons test. (C-D) Correlation analysis of age and CD244 (C), CD160 (D) expression on CD4+ T cells from all healthy individuals. Spearman's non-parametric test was used to test for correlations. $*P < 0.05$.

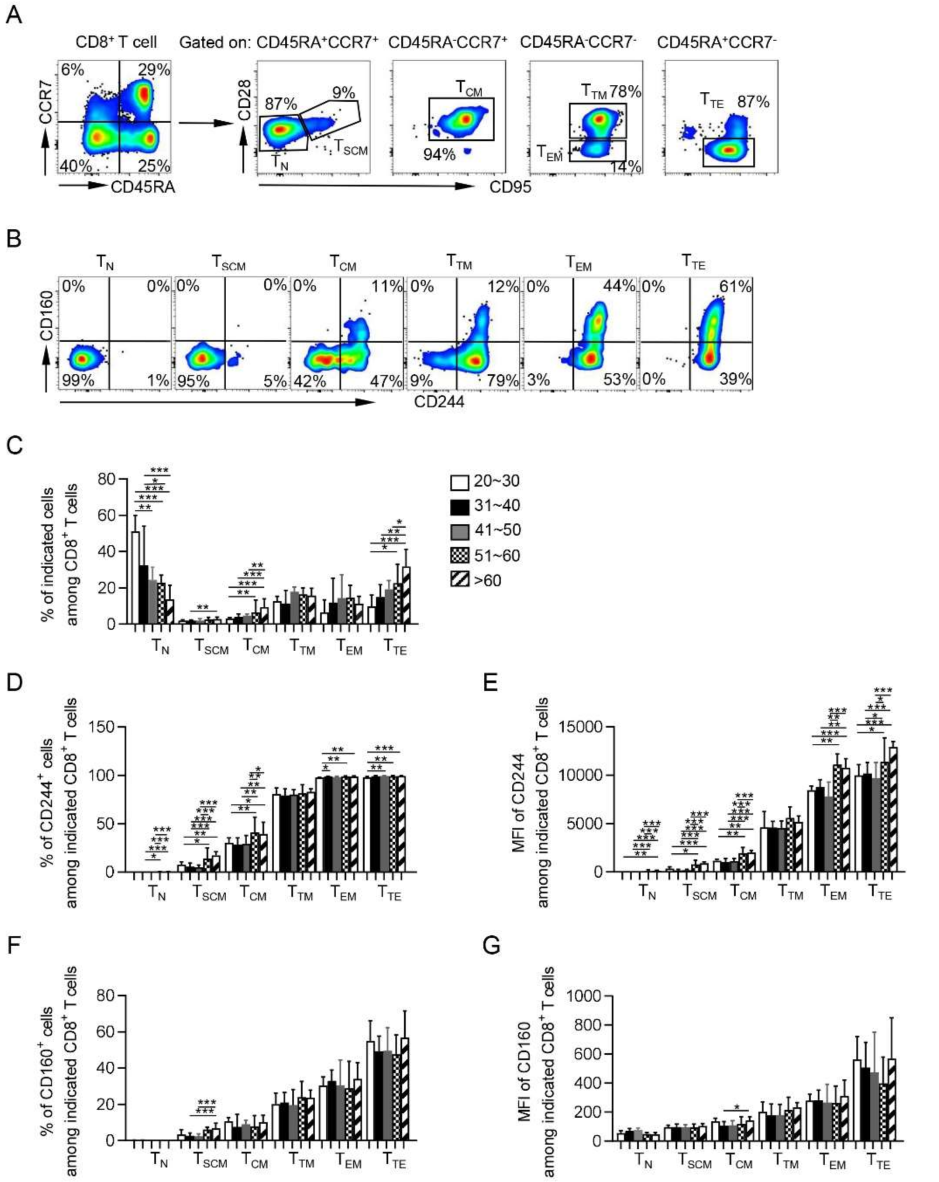

Supplementary Figure 2. Distribution of CD8$^+$ T-cell subsets and CD244, CD160 expression on CD8$^+$ T-cell subsets from different age groups

(A-B) Gating strategy for CD244 and CD160 expression on each subset (T_N, T_{SCM}, T_{CM}, T_{TM}, T_{EM} and T_{TE}) of CD8$^+$ T cells. (C) Distribution of T_N, T_{SCM}, T_{CM}, T_{TM}, T_{EM} and T_{TE} in CD8$^+$ T cells from different age groups (n = 17-34 each group). *P* values were obtained by Kruskal-Wallis test followed by Dunn's multiple comparisons test (T_N, T_{SCM}, T_{CM}, T_{TM} and T_{EM}) or one-way ANOVA followed by Tukey's multiple comparisons test (T_{TE}). (D-E) Expression of CD244 on each subset (T_N, T_{SCM}, T_{CM}, T_{TM}, T_{EM} and T_{TE}) of CD8$^+$ T cells (n = 17-34 each group). *P* values were obtained by Kruskal-Wallis test followed by Dunn's multiple comparisons test (T_N, T_{SCM}, T_{TM}, T_{EM} and T_{TE} in D, T_N, T_{SCM}, T_{TM}, and T_{EM} in E) or one-way ANOVA followed by Tukey's multiple comparisons test (T_{CM} in D, T_{CM} and T_{TE} in E). (F-G) Expression of CD160 on each subset (T_N, T_{SCM}, T_{CM}, T_{TM}, T_{EM} and T_{TE}) of CD8$^+$ T cells (n = 17-34 each group).

P values were obtained by Kruskal-Wallis test followed by Dunn's multiple comparisons test (T_{N}, T_{SCM}, T_{CM} and T_{TM} in F, T_{SCM}, T_{TM}, T_{EM} and T_{TE} in G) or one-way ANOVA followed by Tukey's multiple comparisons test (T_{EM} and T_{TE} in F, T_{N} and T_{CM} in G). $^{*}P < 0.05$, $^{**}P < 0.01$, $^{***}P < 0.001$.

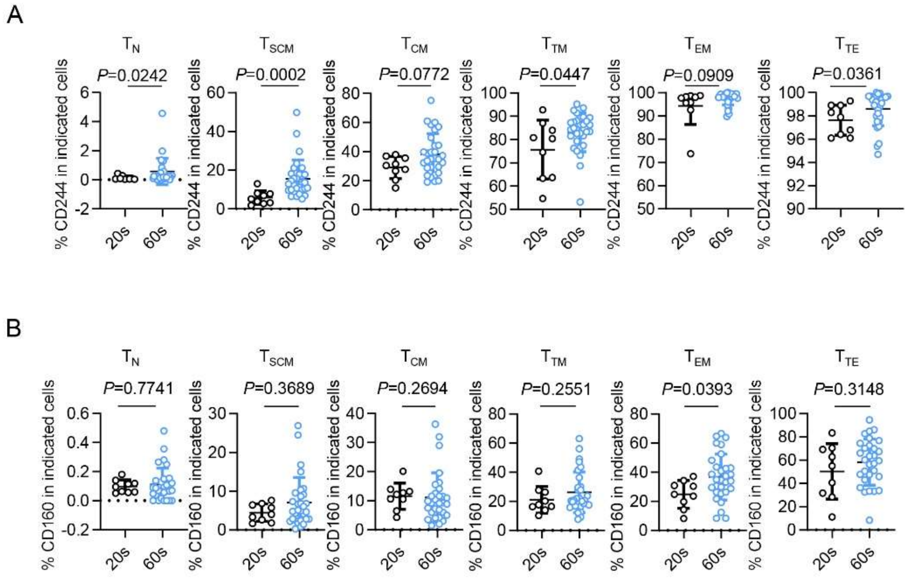

Supplementary Figure 3. **Expression of CD244 and CD160 on CD8$^+$ T cell subsets from young (20s) and elderly (60s)**

(A-B) Scatter dot plots of the expression of CD244 and CD160 cells on indicated CD8$^+$ T cell subsets from healthy donors in 20s and 60s (n = 9-30 each group). *P* values were obtained by Mann-Whitney test or Unpaired t test.

Supplementary Tables

Supplementary Table 1. The sequence of primers for metabolic-related genes.

Genes	Forward primer (5’→3’)	Reverse primer (5’→3’)
GLUT1	5′-TTGGCTCCGGTATCGTCAAC-3′	5′-GCCAGGACCCACTTCAAAGA-3′
HK2	5′-GATTTCACCAAGCGTGGACT-3′	5′-ACAGGTGCTCTCAAGCCCTA-3′
ENO1	5′-TCATCAATGGCGGTTCTCA-3′	5′-TTCCCAATAGCAGTCTTCAGC-3′
PDK1	5′-CTGTGATACGGATCAGAAACCG-3′	5′-TCCACCAAACAATAAAGAGTGCT-3′
ATP5G1	5′-ACAGCAACTTCCCACTCCAG-3′	5′-GCCAAGAATGGCATAGGAGA-3′
mtNd1	5′-GGCTATATACAACTACGCAAAGGC-3′	5′-GGTAGATGTGGCGGGTTTTAGG-3′
β-actin	5′-GAGCTACGAGCTGCCTGACG-3′	5′-GTAGTTTCGTGGATGCCACAG-3′

Supplementary Table 2. Antibody List.

Reagent	Source	Reference number (Cat#)
Anti-human CD3-BV786 (SK7)	BD Pharmingen	563800
Anti-human CD4-APC-Fire750 (SK3)	BD Pharmingen	344638
Anti-human CD8-BV421 (SK1)	BD Pharmingen	740093
Anti-human CD244-PE-D594 (2-69)	BD Pharmingen	564881
Anti-human CD160-AF488 (BY55)	BD Pharmingen	562351
Anti-human CD98-BUV395 (UM7F8)	BD Pharmingen	744508
Anti-human CD45RA-AF700 (HI100)	BD Pharmingen	560673
Anti-human CD57-BV421 (NK-1)	BD Pharmingen	563896
Anti-human PD-1-BV711 (EH12.1)	BD Pharmingen	564017
Anti-human TIM-3-BV650 (7D3)	BD Pharmingen	565564
Anti-human Granzyme B-AF700 (GB11)	BD Pharmingen	560213
Anti-human T-bet-BV421 (O4-46)	BD Pharmingen	563318
Anti-human TNF-α BV711(MAb11)	BioLegend	502943
Anti-human IL-2-BV650 (MQ1-17H12)	BioLegend	500334
Anti-human CCR7-BV421 (G043H7)	BioLegend	353208

Anti-human HLA-DR-AF700 (L243)	BioLegend	307626
Anti-human CD38-BV421 (HB-7)	BioLegend	356618
Anti-human CD28-BV711 (CD28.2)	BioLegend	302948
Anti-human CD27-BV650 (O323)	BioLegend	302828
Anti-human KLRG-1-APC-Fire750 (SA231A2)	BioLegend	367718
Anti-human Annexin V-APC	BioLegend	640920
Anti-human Ki67-BV711 (Ki-67)	BioLegend	350516
Anti-human perforin-APC (B-D48)	BioLegend	353312
Anti-human CD71-FITC (CY1G4)	BioLegend	334104
Anti-human CD107a BV421 (H4A3)	BioLegend	328626
Anti-human Eomes-PE-CY7 (WD1928)	Ebioscience	25-4877-42
Fixable Viability Dye eFluor™ 506	Ebioscience	65-0866-14
Anti-human TIGIT-PE-Cy7 (MBSA43)	Ebioscience	25-9500-42
Anti-human LAG-3-APC (3DS223H)	Ebioscience	17-2239-42
Anti-human 2-NBDG	Ebioscience	N13195
Anti-human IFN-γ-AF700 (4S.B3)	Ebioscience	56-7319-42
Human CD244 (999602)	R&D systems	MAB10393-100
Human CD160 (688327)	R&D systems	MAB6700
Mouse IgG 2B isotype control (688327)	R&D systems	MAB004
Cell Event™ Senescence Green	Thermo Fisher Scientific	C10841
